# A distance-based model for convergent evolution

**DOI:** 10.1007/s00285-023-02038-9

**Published:** 2024-01-18

**Authors:** Barbara Holland, Katharina T. Huber, Vincent Moulton

**Affiliations:** 1 School of Natural Sciences, University of Tasmania, ARC Centre of Excellence for Plant Success, Hobart, Tasmania Australia; 2 School of Computing Sciences, University of East Anglia, Norwich Research Park, Norwich, NR4 7TJ Norfolk UK

**Keywords:** Convergent evolution, Ultrametric, Equidistant tree, Tree metric, Triplet respecting metric, 92-10, 92B10, 92D15, 05C05, 05C12

## Abstract

Convergent evolution is an important process in which independent species evolve similar features usually over a long period of time. It occurs with many different species across the tree of life, and is often caused by the fact that species have to adapt to similar environmental niches. In this paper, we introduce and study properties of a distance-based model for convergent evolution in which we assume that two ancestral species converge for a certain period of time within a collection of species that have otherwise evolved according to an evolutionary clock. Under these assumptions it follows that we obtain a distance on the collection that is a modification of an ultrametric distance arising from an equidistant phylogenetic tree. As well as characterising when this modified distance is a tree metric, we give conditions in terms of the model’s parameters for when it is still possible to recover the underlying tree and also its height, even in case the modified distance is not a tree metric.

## Introduction

One of the central questions in the area of phylogenetics is to develop models and algorithms to reconstruct the evolutionary history of a set of species (or taxa) in terms of a *phylogenetic tree* (Felsenstein 2004). Typically, this is an edge-weighted, rooted tree whose leaves correspond to the set of species in question. A key evolutionary assumption that underpins most phylogenetic models and phylogenetic tree reconstruction methods is that once a speciation has occurred the child species are then conditionally independent and that they diverge from each other at a fairly constant rate. This assumption implies that pairs of species with an older least common ancestor will be at a greater evolutionary distance from each other—in terms of either their molecular sequences or morphology—than those with a more recent least common ancestor (Zuckerkandl and Pauling 1965).

Even so, there are several biological processes which do not conform to this general rule. For example, in virus evolution, recombination events might introduce large chunks of genetic material from one virus into another (Pérez-Losada et al. 2015), bacterial species are known to exchange genes via horizontal gene transfer Dagan and Martin (2006), and both plant and animal species can exchange genetic material through processes such as hybridisation and introgression (Mallet 2005). In extreme cases this latter process is sufficient to cause “reverse speciation” (Rudman and Schluter 2016), a process that is of increasing interest in conservation genetics (Bohling 2016; Seehausen 2006). These types of evolution are commonly known as *reticulate evolution* and there is a growing body of literature concerning the use of a generalisation of phylogenetic trees called *(rooted) phylogenetic networks* to model and represent such evolutionary scenarios (Bapteste et al. 2013).

Another important evolutionary process which may also cause species to become more alike even in cases where they are not more genetically similar is known as *convergent evolution*. It occurs with many different species across the tree of life, and, in contrast to reticulate evolution, genetic material is not exchanged (see e.g. Sackton and Clark (2019) for a recent review). Convergent evolution usually acts over long periods of time and there has been less attention on modelling this gradual process.

In Sumner et al. (2012), a very general Markov model of character evolution was proposed that allowed species to either diverge or converge according to different partitions of the species set in different epochs. These convergence-divergence models were explored further in Mitchell (2016) and Mitchell et al. (2018) where questions of identifiability and distinguishability of different subclasses of model were addressed (Mitchell 2016; Mitchell et al. 2018). Perhaps unsurprisingly, they found that not all convergence-divergence models could be distinguished from each other or from the standard tree model.

Despite these advances, to our best knowledge no-one has investigated models allowing convergence of species from the perspective of distance data. Evolutionary distances are commonly inferred from species data, for example, from morphological features or molecular sequences, and there are several approaches to reconstruct evolutionary trees from such distances [e.g. (Felsenstein, 2004, Chapter 11)]. Here we are interested in modelling the situation where convergence between species or lineages has occurred over a sustained period and acts so as to reduce the evolutionary distance between species or at least slows down their rate of divergence. In particular, we seek to understand when such processes will leave a discernible trace in distance data, and to characterise the situations where the underlying tree topology is recoverable.

**Fig. 1:**
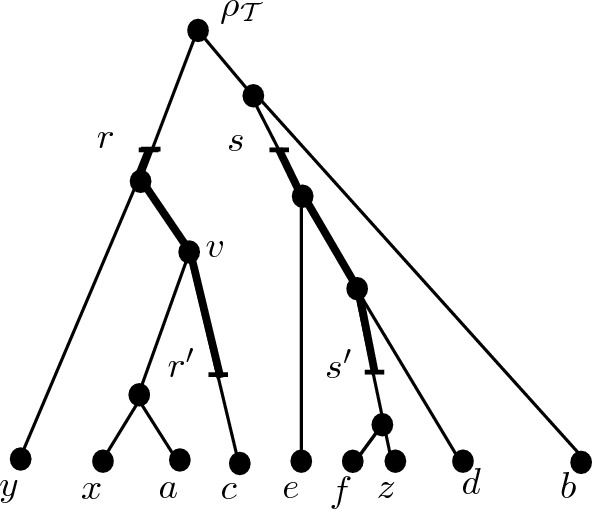
An example of a convergence model on the set of species $X=\{a,b,c,d,e,f,x,y,z\}$ associated to an edge-weighted rooted tree ${\mathcal {T}}$ with root $\rho _{{\mathcal {T}}}$. The weights of the edges are proportional to their lengths. The points *r*, *s* represent two ancestral species which have converged to give rise to the species $r',s'$, respectively, over a period of time that is proportional to the length of the two bold paths

An illustration of our distance-based convergence model is presented in Fig. 1 (see Sect. 3 for precise definitions). We start with an edge-weighted, rooted phylogenetic tree ${\mathcal {T}}$ on a collection *X* of species in which the length of any path from the root $\rho _{{\mathcal {T}}}$ of ${\mathcal {T}}$ to any leaf has the same length (i.e. ${\mathcal {T}}$ is an *equidistant tree* (Semple and Steel 2003)). Such trees are commonly used to represent the evolutionary history of a collection of species that have undergone “clock-like” evolution.

Under this assumption, the evolutionary distance $d_{\mathcal T}(x,y)$ between any pair of species $x,y \in X$ is given by taking the length of the shortest path between *x* and *y* in ${\mathcal {T}}$, so that $d_{{\mathcal {T}}}(x,y)$ is proportional to twice the time that has passed since the last common ancestor of *x* and *y* speciated. To model convergence, we assume that at some time in the past two ancestral species (or lineages), represented by the two points *r*, *s* in ${\mathcal {T}}$ at the same distance to $\rho _{\mathcal T}$, have been subject to convergence for a certain period of time. We represent this by two equal-length and disjoint paths in ${\mathcal {T}}$ (represented in bold) that start at *r* and *s* and end at two points $r'$ and $s'$, respectively. In particular, we are also assuming that $r'$ and $s'$ will diverge from one another after the convergence period has ended.

Using the information given by this model we adjust the distance $d_{{\mathcal {T}}}$ on *X* to obtain a new distance $d'_{{\mathcal {T}}}$ on *X* as follows. For any $x,y \in X$ that lie below the points *r* and *s* we subtract a certain amount off of the distance $d_{{\mathcal {T}}}(x,y)$ that is proportional to the period of time that the ancestors of *x* and *y* have undergone convergence as determined by the two disjoint paths. Note that the distance $d'_{{\mathcal {T}}}$ should be thought of as being a distance that is inferred directly from the set of species *X*. These distances could, for example, be given by computing some distance between molecular sequences representing species in *X*, morphological data, or broader genomic features such as gene presence/absence. The mathematical aim is to then understand when we can recover $\mathcal T$ from $d'_{{\mathcal {T}}}$. Interestingly, as we shall see, for our model of convergence even though $d'_{{\mathcal {T}}}$ will no longer necessarily correspond to an equidistant tree, it may still be possible to recover the topology of ${\mathcal {T}}$ from $d'_{\mathcal T}$, even in case $d'_{{\mathcal {T}}}$ does not correspond to any tree.

We now summarise the contents of the rest of this paper. In Sect. 2, we present some basic definitions and facts concerning phylogenetic trees, ultrametrics and tree metrics. In Sect. 3, we present our model of convergence and characterise when the map $d'_{{\mathcal {T}}}$ above is in fact a distance (Lemma 1). In Sect. 4, we then focus on the question of how to recover the topology of ${\mathcal {T}}$ from $d'_{{\mathcal {T}}}$. To do this we consider three-leaved subtrees of ${\mathcal {T}}$ called *triplets*, and characterise when $d'_{{\mathcal {T}}}$ is *triplet respecting*, that is, when it is possible to recover the triplets of *T* (and hence the topology of ${\mathcal {T}}$) from $d'_{{\mathcal {T}}}$ (Theorem 3). In Sect. 5, under the assumption that $d'_{{\mathcal {T}}}$ is triplet-respecting, we characterise when $d'_{{\mathcal {T}}}$ is either an ultrametric or a tree metric (Theorem 8). In Sect. 6, we then focus in when it is possible to also recover the height of ${\mathcal {T}}$ from $d'_{{\mathcal {T}}}$ in case we are able to recover ${\mathcal {T}}$ from $d'_{{\mathcal {T}}}$ (Theorem 11). We conclude in Sect. 7 with a brief discussion of possible future directions.

## Preliminaries

In this paper, *X* is a finite set (of species or taxa) and $n=|X|$. We assume $n \ge 3$.

A *distance (on X)* is a function $d:X \times X \rightarrow \mathbb R_{\ge 0}$ such that, for all $x,y \in X$, $d(x,y)=0$ if and only if $x=y$, and $d(x,y)=d(y,x)$ (i.e. *d* is *symmetric*). A distance *d* on *X* is

- a *metric* if for all distinct $x,y,z \in X$, $d(x,y) \le d(x,z) + d(z,y)$;
- a *tree metric* if it satisfies the *four-point condition, i. e. *for all (not necessarily distinct) $x,y,u,v\in X$,
  1 $$\begin{aligned} d(x,y)+d(u,v) \le \max \{d(x,u)+d(y,v), d(x,v)+ d(y,u)\}; \end{aligned}$$
- an *ultrametric* if for all distinct $x,y,z \in X$,
  2 $$\begin{aligned} d(x,y) \le \max \{d(x,z),d(y,z)\}. \end{aligned}$$

Note that an ultrametric is a tree metric, a tree metric is a metric, and that there are tree metrics that are not ultrametrics, metrics that are not tree metrics and distances that are not metrics. Also note that if *d* is a metric on *X*, then *d* is a tree metric if and only if *d* satisfies the 4-point condition for every *pairwise distinct* $x,y,u,v\in X$.

A *(binary) phylogenetic tree* *T* (*on* *X*) is a rooted tree, with root $\rho = \rho _T$ and leaf-set *X* such that the degree of $\rho$ is two and the degree of any other non-leaf vertex in *T* is three. An *edge-weighted* phylogenetic tree $\mathcal T = (T,w)$ is a phylogenetic tree $T=(V,E)$ together with a weight function $w: E \rightarrow {\mathbb {R}}_{> 0}$, which assigns a positive weight to each edge in *T*. To a phylogenetic tree *T* on *X*, we associate the distance $d_{{\mathcal {T}}}=d_{(T,w)}$ on *X* given by, for $x,y\in X$, setting $d_{{\mathcal {T}}}(x,y)$ equal to the length of the path in *T* between *x* and *y* (i.e. the sum of the edge-weights taken over the edges in the path in *T* between *x* and *y*). Note that $d_{{\mathcal {T}}}$ is necessarily a tree metric.

We shall also consider an edge-weighted phylogenetic tree $\mathcal T=(T,w)$ as being a continuous object, that is, we consider an edge *e* of *T* with weight *w*(*e*) as being a real, closed interval with length *w*(*e*). In particular, we will consider a *point* in *T* to be an element in some edge of *T*. Note that vertices in *T* are considered as points in *T*, and that we will use the terms vertex and point interchangeably when it is clear what we mean from the context. When we want to emphasise that a point is not a vertex we shall say that it is *inside* of an edge. Note that we have a natural ordering of the points in ${\mathcal {T}}$. Given two points *a*, *b* in *T*, we say that *a* is *above* *b* (or *b* is *below* *a*) if either $a=b$ or the path from the root of *T* to *b* (thought of as a continuous object) contains *a*; if *b* is below *a* and not equal to *a* we say that *b* is *strictly* below *a*. Moreover, we define the *least common ancestor* $lca(a,b)=lca_{T}(a,b)$ of *a* and *b* to be the lowest point in *T* that is above both *a* and *b*. Note that $lca(a,a)=a$.

An *equidistant* tree is an edge-weighted phylogenetic tree ${\mathcal {T}}=(T,w)$ such that for any two leaves *x* and *y* of *T* the length of the path in ${\mathcal {T}}$ from the root $\rho$ of *T* to *x* equals the length of the path in ${\mathcal {T}}$ from $\rho$ to *y*. Given such a tree ${\mathcal {T}}$, the *height* $h(a)=h_{{\mathcal {T}}}(a)$ of a point *a* of ${\mathcal {T}}$ is the length of the path in ${\mathcal {T}}$ from *a* to any leaf below *a*. We refer to the height of the root of *T* as the *height* of ${\mathcal {T}}$. We call an equidistant tree *generic* if for any pair *v*, *w* of distinct non-leaf vertices in *T*, $h(v)\ne h(w)$.

## Convergence scenarios

**Fig. 2:**
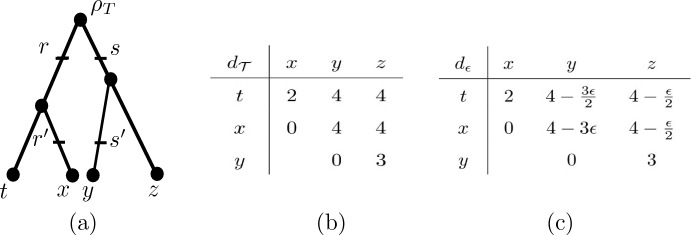
**a** Example of a convergence scenario $(\mathcal T=(T,w),R,\epsilon )$ on $X=\{x,y,z,t\}$, where *T* is the depicted phylogenetic tree on *X*, $h(\rho _T)=2$, $h(lca_{T}(t,x))=1$, $h(lca_{T}(y,z))=\frac{3}{2}$, $\alpha =\frac{1}{4}$, $\beta =\frac{7}{4}$, and $0< \epsilon < \frac{4}{3}$. **b** The distance matrix for $d_{{\mathcal {T}}}$. **c** The distance matrix $d_{\epsilon }$. Note that $d_{\epsilon }$ is a metric, but not a tree metric

In this section, we formally define our convergence model which is based on the following parameters: (1) a real non-negative number $\epsilon \ge 0$, (2) a generic equidistant tree ${\mathcal {T}}= (T,w)$ on *X* with height $h>0$, (3) two non-negative numbers $\alpha$ and $\beta$ with $0< \alpha< \beta < h$, and $\alpha , \beta$ not equal to the height of any vertex in *T*, and (4) a *convergence set* *R* in ${\mathcal {T}}$, that is, a set of four distinct points say $r,r',s,s'$, each one of them inside some edge of *T*, such *r*, *s* have height $\beta$, the points $r',s'$ have height $\alpha$, the point $r'$ is below *r*, and the point $s'$ is below *s*. We call a triple $({\mathcal {T}},R,\epsilon )$ consisting of some choice of these parameters a *convergence scenario (on X)* (see Fig. 2 for an example). From now on, ${\mathcal {T}}=(T,w)$, $\alpha$, $\beta$, and $\epsilon$ will be as described above.

We now define some additional terminology for convergence scenarios. For such a scenario $({\mathcal {T}},R,\epsilon )$ we call the points in *R* with height $\beta$ the *top points* of *R* and the points with height $\alpha$ the *bottom points* of *R*. We define $lca(R)=lca_{T}(R)$ to be the least common ancestor of the top points of *R* (which is necessarily a vertex in *T*). We say that two distinct elements *x*, *y* in *X* are *(strictly below) below R* if *x* is below one (bottom) top point of *R* and *y* is below the other (bottom) top point of *R*. In addition, given two elements *x*, *y* in *X* below *R*, we define

$$\begin{aligned} h_R(x,y) = \max \{h(lca_{T}(r',x)),h(lca_{T}(s',y))\}, \end{aligned}$$

where $r'$ (respectively $s'$) is the bottom element in *R*, so that *x* and $r'$ (respectively $s'$ and *y*) are both below the same top point in *R*. In addition, we associate a map $d_{\epsilon }=d^{({\mathcal {T}},R)}_{\epsilon }:X \times X \rightarrow {{\mathbb {R}}}$ to $({\mathcal {T}},R,\epsilon )$ as follows. Let $x,y \in X$. If *x*, *y* are below *R* (so that they are necessarily distinct), then set

$$\begin{aligned} d_{\epsilon }(x,y) = d_{{\mathcal {T}}}(x,y) - 2 \epsilon (\beta - h_R(x,y) ), \end{aligned}$$

else set $$d_{\epsilon }(x,y)=d_{{\mathcal {T}}}(x,y)$$.

To help illustrate these concepts, consider the convergence scenario pictured in Fig. 2. Then *t* and *z* are below *R*, but not strictly below *R*, whereas *x* and *y* are strictly below *R*. Furthermore, $h(lca_{{\mathcal {T}}}(r',t))=1<\frac{3}{2}=h(lca_{{\mathcal {T}}}(s',z))$ and $h(lca_{{\mathcal {T}}}(r',x))=\alpha =h(lca_{{\mathcal {T}}}(s',y))$. Hence, $h_R(t,z)=\frac{3}{2}$ and $h_R(x,y)=\alpha$. Finally, since $d_{{\mathcal {T}}}(t,z)=4=d_{{\mathcal {T}}}(x,y)$ we obtain, for example, $d_{\epsilon }(t,z) =4-2\epsilon (\beta -\frac{3}{2})=4- \frac{3}{2}\epsilon +\epsilon =4-\frac{\epsilon }{2}$ and $d_{\epsilon }(x,y)=4-2\epsilon (\beta -\alpha )=4-3\epsilon$. Since *y* and *z* are not both below *R*, we obtain $d_{\epsilon }(y,z)=d_{{\mathcal {T}}}(y,z)=3$.

Note that $d_{\epsilon } \le d_{{\mathcal {T}}}$, $d_{\epsilon }$ is symmetric, and that $d_{\epsilon }$ may take on negative values [i.e., it is what is commonly called a *dissimilarity map* (Semple and Steel 2003)]. Loosely speaking, we can interpret the map $d_{\epsilon }$ as follows. For any two taxa *x* and *y*, the quantity $d_{{\mathcal {T}}}(x,y)$ is proportional to the time that *x* and *y* have diverged from one another. We then subtract $2 \epsilon (\beta - h_R(x,y))$ from this quantity to model the fact that some ancestors of *x* and *y* have converged for a period of time that is proportional to the quantity $\beta - h_R(x,y)$.

In general, given $d_{\epsilon }$, we are interested in recovering the topology of the phylogenetic tree *T* that gives rise to $d_{\epsilon }$. Since in real applications $d_{\epsilon }$ will be non-negative (e.g., it could be a distance matrix computed from a multiple sequence alignment), in rest of this paper we shall focus on the case where $d_{\epsilon }$ is a distance. We conclude this section by giving a characterisation for when this is the case.

### Lemma 1

Let $({\mathcal {T}},R,\epsilon )$ be a convergence scenario. Then $d_{\epsilon }$ is a distance if and only if

$$\begin{aligned} \epsilon < \frac{h(lca(R))}{(\beta - \alpha )}. \end{aligned}$$

### Proof

Suppose $\epsilon < \frac{h(lca(R))}{(\beta - \alpha )}$. Since $d_{{\mathcal {T}}}$ is a distance on *X*, to show that $d_{\epsilon }$ is a distance it clearly suffices to show that $d_{\epsilon }(x,y) > 0$ for all $x,y\in X$ below *R*. Let $x,y \in X$ be below *R*. Then $h_R(x,y) \ge \alpha$, and so

$$\begin{aligned} d_{\epsilon }(x,y)= & {} d_{{\mathcal {T}}}(x,y) - 2 \epsilon ( \beta - h_R(x,y)) = 2h(lca(R)) - 2 \epsilon ( \beta - h_R(x,y)) \\\ge & {} 2[h(lca(R))- \epsilon ( \beta -\alpha )] > 0. \end{aligned}$$

Conversely, if $d_{\epsilon }$ is a distance, then pick some $x,y \in X$ distinct and strictly below *R*. Then $d_{\epsilon }(x,y)>0$. If *x* is below the bottom point $r' \in R$ and *y* is below the bottom point $s' \in R$, it follows that $h(lca_{{\mathcal {T}}}(r',x))=h(lca_{T}(s',y))=\alpha$. Hence, $h_R(x,y)=\alpha$ and so

$$\begin{aligned} 0 < d_{\epsilon }(x,y) = d_{{\mathcal {T}}}(x,y) - 2 \epsilon ( \beta -h_R(x,y)) = 2h(lca(R)) - 2\epsilon ( \beta -\alpha ), \end{aligned}$$

which implies $$\epsilon < h(lca(R))/(\beta - \alpha )$$. $$\square $$

Note that there exist convergence scenarios $({\mathcal {T}},R,\epsilon )$ for which $d_{\epsilon }$ is a distance but not a metric (see for example Sect. 5, Fig. 4(8) where $h(\rho _T)=10$, $h(lca_{T}(x,y))=1$, $h(lca_{T}(x,z))=\frac{1}{5}$, $\beta =\frac{3}{4}$, $\alpha =\frac{1}{4}$, and $\epsilon =5$). There does not appear to be a simple characterisation along the lines of Lemma 1 for when $d_{\epsilon }$ is a metric, although in Sect. 5 we shall give a characterisation for when $d_{\epsilon }$ is a metric in case $d_{\epsilon }$ enjoys some additional properties.

## Recovering the topology of the tree from a convergence scenario

In this section, given a convergence scenario $({\mathcal {T}},R,\epsilon )$ with $d_{\epsilon }=d^{({\mathcal {T}},R)}_{\epsilon }$ a distance, we are interested in understanding when we can recover the topology of ${\mathcal {T}}$ from $d_{\epsilon }$.

To this end, we begin by recalling some useful facts concerning phylogenetic trees. A *triplet* is a phylogenetic tree with three leaves. If *x*, *y*, *z* are the leaves of a triplet, and the least common ancestor of *x* and *y* in the triplet is not the root of the triplet, then we denote the triplet by ((*x*, *y*), *z*). Given a phylogenetic tree *T* with leaf set *X*, we can induce a triplet on every subset of *X* of size three by simply taking the tree spanned by the leaves in this subset and suppressing all non-root vertices contained in precisely two edges. We let ${{\mathcal {R}}}(T)$ denote the set of triplets on *X* induced by *T* in this way. Note that ${{\mathcal {R}}}(T)$ completely determines *T* (Semple and Steel 2003, Theorem 6.4.1) (i.e. there is no phylogenetic tree $T'$ on *X* different from *T* with ${{\mathcal {R}}}(T')={{\mathcal {R}}}(T)$).

Now, for the convergence scenario $({\mathcal {T}},R,\epsilon )$, we associate a set of triplets to $d_{\epsilon }$ by putting

$$\begin{aligned} {{\mathcal {R}}}(d_{\epsilon }) = \{((x,y),z) :\, \{x,y,z\} \in {X \atopwithdelims ()3} \text{ and } d_{\epsilon }(x,y) < \min \{d_{\epsilon }(x,z), d_{\epsilon }(y,z) \} \}. \end{aligned}$$

In case $\epsilon =0$, we have ${{\mathcal {R}}}(d_{\epsilon })= {{\mathcal {R}}}(T)$ but ${{\mathcal {R}}}(d_{\epsilon })= {{\mathcal {R}}}(T)$ need not hold in general. For example, ${{\mathcal {R}}}(d_{\epsilon })\ne {{\mathcal {R}}}(T)$ for the convergence scenario pictured in Fig. 2 for $\epsilon =1$ because $((x,y),z)\in {\mathcal {R}}(d_{\epsilon })- {\mathcal {R}}(T)$. Motivated by this, we say that $d_{\epsilon }$ is *triplet respecting* if ${{\mathcal {R}}}(d_{\epsilon }) = {{\mathcal {R}}}(T)$. It follows that if $d_{\epsilon }$ is triplet respecting, then we can recover *T* from ${{\mathcal {R}}}(d_{\epsilon })$ using, for example, the Build algorithm (Semple and Steel 2003).

Interestingly, there are convergence scenarios $({\mathcal {T}},R,\epsilon )$ where $d_{\epsilon }$ is a distance that is *not* a tree metric (and therefore not an ultrametric), but where $d_{\epsilon }$ is still triplet respecting (e.g. in Fig. 2 take any $\epsilon$ with $0<\epsilon < \frac{1}{3}$). Thus in some cases we can recover the tree *T* from $d_{\epsilon }$ even though $d_{\epsilon }$ is not a tree metric. Hence, it is of interest to characterise when $d_{\epsilon }$ is a triplet respecting distance.

To this end, we begin with a useful but somewhat technical observation. Given a convergence scenario $({\mathcal {T}},R,\epsilon )$ on *X*, we associate a convergence scenario to each triple $Y= \{x,y,z\} \subseteq X$, for which $((x,y),z) \in {\mathcal {R}}(T)$ and $d_{\epsilon }|_Y \ne d_{{\mathcal {T}}}|_Y$ (i.e. $d_{\epsilon }(a,b) \ne d_{{\mathcal {T}}}(a,b)$ for some $a,b \in Y$) as follows.

**Fig. 3:**
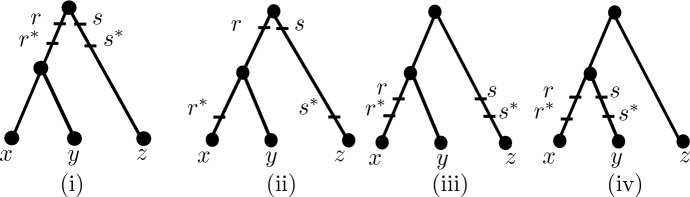
The configurations used within the proof of Theorem 3

Let ${\mathcal {Q}}$ denote the edge-weighted triplet ((*x*, *y*), *z*) whose edge weighting is induced by the edge-weighting of *T* (so that ${\mathcal {Q}}$ is an edge-weighted phylogenetic tree on *Y*). Note that since $d_{\epsilon }|_Y \ne d_{{\mathcal {T}}}|_Y$, at least two elements in *Y* must be below *R*, and so the top points in *R* are contained in ${\mathcal {Q}}$. Even so, the bottom points of *R* will not necessarily be contained in ${\mathcal {Q}}$. However, by interchanging the roles of *x* and *y* if necessary, we obtain a triple $({\mathcal {Q}},R^*=\{r,s,r^*,s^*\},\epsilon )$ as pictured in one of Fig. 3i–iv by giving labels *r* and *s* to the top points of *R* and giving the points $r^*$ and $s^*$ height equal to $\max \{h_{r'},h_{s'},\alpha \}$, where $h_{r'}$ and $h_{s'}$ are the heights of the points where the paths from $r'$ and $s'$ to *lca*(*R*) join ${\mathcal {Q}}$, respectively. We refer to $({\mathcal {Q}},R^*,\epsilon )$ as the *restriction of* $({\mathcal {T}},R,\epsilon )$ *to Y*. For example, for the triplet ((*x*, *y*), *z*) coming from the phylogenetic tree in Fig. 1, we would obtain the convergence scenario as in Fig. 3ii, where $r^*$ and $s^*$ have height equal to the height of the vertex *v* in Fig. 1 (since $r^*=v$, $s^*=s'$ and *v* is higher than $s'$).

The proof of the following is routine case checking, and so we omit it.

### Lemma 2

Let $({\mathcal {T}},R,\epsilon )$ be a convergence scenario on *X* and let $Y=\{x,y,z\}$ be a triple of distinct elements in *X* such that $((x,y),z) \in {\mathcal {R}}(T)$ and $d_{\epsilon }|_Y \ne d_{{\mathcal {T}}}|_Y$. Then the triple $({\mathcal {Q}}, R^*, \epsilon )$ defined in the preceding paragraph is a convergence scenario on *Y*. Moreover, $d_{\epsilon }|_Y= d_{\epsilon }^{({\mathcal {Q}}, R^*)}$.

We call a convergence scenario $({\mathcal {T}},R,\epsilon )$ a *cherry scenario* if the points in *R* are all contained in two edges of some *cherry* in *T*, that is, two leaves in *T* that are adjacent to a common vertex.

### Theorem 3

Suppose that $({\mathcal {T}},R,\epsilon )$ is a convergence scenario on *X* such that $d_{\epsilon }$ is a distance. If $({\mathcal {T}},R,\epsilon )$ is a cherry scenario, then $d_{\epsilon }$ is triplet respecting, else $d_{\epsilon }$ is triplet respecting if and only if for all distinct $x,z \in X$ strictly below *R* and $y \in X$ such that $((x,y),z) \in {\mathcal {R}}(T)$,

3 $$\begin{aligned} \epsilon < \frac{d_{{\mathcal {T}}}(x,z)-d_{{\mathcal {T}}}(x,y)}{2(\beta -\alpha )}. \end{aligned}$$

### Proof

Put $d_{\epsilon }=d_{\epsilon }^{({\mathcal {T}}, R)}$ and $d_{\epsilon }^*=d_{\epsilon }^{({\mathcal {Q}}, R^*)}$. If $({\mathcal {T}}=(T,w),R,\epsilon )$ is a cherry scenario, then it is straight-forward to see that $d_{\epsilon }$ is triplet respecting.

Now, suppose that $({\mathcal {T}},R,\epsilon )$ is not a cherry scenario. Suppose first that $d_{\epsilon }$ is triplet respecting, so that ${{\mathcal {R}}}(d_{\epsilon }) = {{\mathcal {R}}}(T)$ holds. Suppose $x,z \in X$ are strictly below *R* and $y \in X$ is such that $((x,y),z) \in {{\mathcal {R}}}(T)$. Then, $d_{\epsilon }(x,y)=d_{{\mathcal {T}}}(x,y)$, $d_{\epsilon }(x,z) \le d_{\epsilon }(y,z)$ and $d_{\epsilon }(x,z)= d_{{\mathcal {T}}}(x,z) -2\epsilon (\beta - \alpha )$. Moreover, since ${{\mathcal {R}}}(d_{\epsilon }) = {{\mathcal {R}}}(T)$, we must have $d_{\epsilon }(x,y) < \min \{d_{\epsilon }(x,z),d_{\epsilon }(y,z)\}$. Hence,

$$\begin{aligned} d_{{\mathcal {T}}}(x,y)= d_{\epsilon }(x,y) <d_{\epsilon }(x,z)= d_{{\mathcal {T}}}(x,z) -2\epsilon (\beta - \alpha ), \end{aligned}$$

which implies that Inequality (3) holds.

Conversely, suppose that $((x,y),z) \in {\mathcal {R}}(T)$ that *x*, *z* are strictly below *R* and that Inequality (3) holds. We will show that $d_{\epsilon }(x,y) < \min \{d_{\epsilon }(x,z), d_{\epsilon }(y,z)\}$, from which it follows that ${\mathcal {R}}(d_{\epsilon })={\mathcal {R}}(T)$ (i.e. $d_{\epsilon }$ is triplet respecting). Put $Y=\{x,y,z\}$. If $d_{\epsilon }|_Y = d_{{\mathcal {T}}}|_Y$, then the inequality clearly holds. So, suppose $d_{\epsilon }|_Y \ne d_{{\mathcal {T}}}|_Y$. Then consider the convergence scenario $({\mathcal {Q}},R^*,\epsilon )$ that is obtained by restricting ${\mathcal {T}}$ to *Y* which, without loss of generality, must be as in one of the configurations in Fig. 3i–iv. By Lemma 2$d_{\epsilon }|_Y= d_{\epsilon }^*$, and so considering each of the cases (i)–(iv) in Fig. 3 we have $d_{\epsilon }(x,y) < \min \{d_{\epsilon }(x,z), d_{\epsilon }(y,z)\}$ since in (i) by (3)

$$\begin{aligned} d_{\epsilon }(x,y) =d_{{\mathcal {T}}}(x,y) <d_{{\mathcal {T}}}(x,z)- 2\epsilon (\beta -\alpha )= d_{\epsilon }(x,z) \end{aligned}$$

and $d_{{\mathcal {T}}}(y,z)=d_{{\mathcal {T}}}(x,z)$ implies $d_{\epsilon }(x,z)= d_{\epsilon }(y,z)$; in (ii) using similar reasoning to case (i), $d_{\epsilon }(x,y) = d_{{\mathcal {T}}}(x,y) < d_{\epsilon }(x,z) \le d_{\epsilon }(y,z)$; in (iii) using similar reasoning to case (i) again, $d_{\epsilon }(x,y) < d_{\epsilon }(x,z) \le d_{\epsilon }(y,z)$, and in (iv) by (3)

$$\begin{aligned} d_{\epsilon }(x,y)<d_{{\mathcal {T}}}(x,y) <d_{{\mathcal {T}}}(x,z)=d_{\epsilon }(x,z), \end{aligned}$$

and $$d_{\epsilon }(y,z)=d_{{\mathcal {T}}}(y,z)=d_{{\mathcal {T}}}(x,z)$$. $$\square $$

As a consequence of Theorem 3, we immediately obtain the following simple condition which guarantees that the distance $d_{\epsilon }$ is triplet respecting.

### Corollary 4

Suppose that $({\mathcal {T}}=(T,w),R,\epsilon )$ is a convergence scenario such that $d_{\epsilon }$ is a distance. If $({\mathcal {T}},R,\epsilon )$ is not a cherry scenario and

$$\begin{aligned} \epsilon <\min \{\frac{w(e)}{\beta -\alpha } :\, e \text{ is } \text{ an } \text{ edge } \text{ in } T \text{ not } \text{ containing } \text{ a } \text{ leaf } \text{ of } T \} \end{aligned}$$

then $d_{\epsilon }$ is triplet-respecting

### Proof

This follows from Theorem 3 since if $x,y,z \in X$ such that *x*, *z* are strictly below *R* and $y\in X$ such that $((x,y),z)\in {\mathcal {R}}(T)$ then $(d_{{\mathcal {T}}}(x,z)-d_{{\mathcal {T}}}(x,y))/2$ is equal to the length of the path in ${\mathcal {T}}$ between *lca*(*R*) and $lca_T(x,y)$ and this path must contain at least one edge in *T* which does not contain a leaf. $\square$

**Fig. 4:**
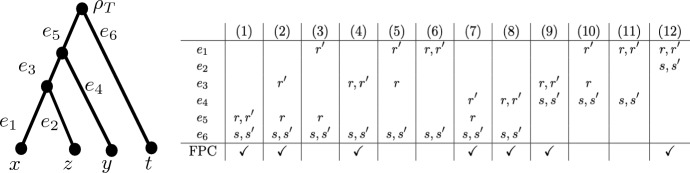
A phylogenetic tree *T*, together with a table that indicates in each column which edges contain the top points *r*, *s* and bottom points $r',s'$ used to form a convergence scenario. For example, column 10 indicates that top point *r* is contained in edge $e_3$, bottom point $r'$ is in edge $e_1$ and points $s,s'$ are both contained in edge $e_4$. The last row indicates whether the configuration gives rise to a distance satisfying the four-point condition or not (see Lemma 7)

Note that the converse of Corollary 4 does not hold. For example, consider the convergence scenario depicted in Fig. 4(10) where $\alpha =\frac{1}{2}$, $\beta =\frac{3}{2}$, $h(lca_{T}(y,z))=2$, $h(lca_{T}(x,z))=1$, and $h(\rho _T)=2+\delta$, for $\delta >0$. Then the bound on $\epsilon$ given in Theorem 3 is 1 (it is given by the three elements *x*, *y*, *z*), and for $\epsilon =\frac{1}{2}$, $d_{\epsilon }$ is a triplet respecting distance. So if the converse of Corollary 4 held, then for $\delta = \frac{1}{1000}$ we would have $\frac{1}{2}= \epsilon < \delta = \frac{1}{1000}$ which is impossible.

## Triplet respecting metrics

**Fig. 5:**
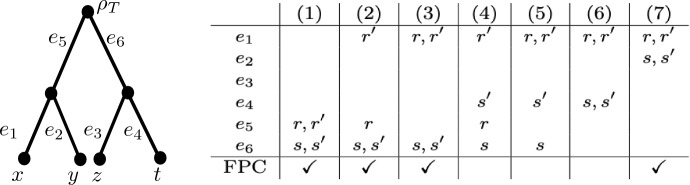
A phylogenetic tree *T*, together with a table that indicates in each column which edges contain the top points *r*, *s* and bottom points $r',s'$ used to form a convergence scenario as in Fig. 4. The last row indicates whether the configuration gives rise to a distance satisfying the four-point condition or not (see Lemma 7)

In this section, in case a convergence scenario gives rise to a triplet respecting distance $d_{\epsilon }$, we want to characterise under which circumstances $d_{\epsilon }$ is a metric, a tree metric or an ultrametric. Note that these are proper subclasses since there are examples of triplet respecting distances $d_{\epsilon }$ where:

1. $d_{\epsilon }$ is a distance but not a metric (in Fig. 4(8) take $h(\rho _T)=10$, $h(lca(x,y))=1$, $h(lca(x,z))=\frac{1}{5}$, $\beta =\frac{3}{4}$, $\alpha =\frac{1}{4}$, and $\epsilon =5$),
2. $d_{\epsilon }$ is a metric but not a tree metric (in Fig. 5(6) take $h(\rho _T)=2$, $h(lca(x,y))=1$, $h(lca(t,z))=1+\delta$, $\beta =\frac{3}{4}$, $\alpha =\frac{1}{4}$, and $\epsilon , \delta >0$ are both small), and
3. $d_{\epsilon }$ is a tree metric but not an ultrametric (in Fig. 4(8) take $h(\rho _T)=3$, $h(lca(x,y))=2$, $h(lca(x,z))=1$, $\beta =1+\delta$, $\alpha =1-\delta$, and $\epsilon , \delta >0$ are both small).

We start by characterising when a triplet respecting distance is a metric.

### Theorem 5

Suppose that $({\mathcal {T}},R,\epsilon )$ is a convergence scenario such that $d_{\epsilon }$ is a triplet respecting distance. Then $d_{\epsilon }$ is a metric if and only if for all distinct $x,z \in X$ strictly below *R* and $y \in X$ such that $((x,y),z) \in {\mathcal {R}}(T)$ and $lca_T(x,y)$ is not below a bottom point of *R*,

4 $$\begin{aligned} \epsilon < \frac{d_{{\mathcal {T}}}(x,y)}{2(\min \{\beta ,h_{{\mathcal {T}}}(lca(x,y))\}-\alpha )}. \end{aligned}$$

### Proof

First note that since $d_{\epsilon }$ is a distance, $d_{\epsilon }$ is a metric if and only if the triangle inequality holds for every triple $Y=\{x,y,z\}$ of distinct elements $x,y,z \in X$, i.e. (A) $d_{\epsilon }(x,y) \le d_{\epsilon }(x,z) + d_{\epsilon }(z,y)$, (B) $d_{\epsilon }(x,z) \le d_{\epsilon }(x,y) + d_{\epsilon }(y,z)$, and (C) $d_{\epsilon }(y,z) \le d_{\epsilon }(y,x) + d_{\epsilon }(x,z)$ all hold.

($\Leftarrow$) Suppose $Y=\{x,y,z\}$ is a triple of elements in *X*. To show that $d_{\epsilon }$ is a metric, we need to show that (A)–(C) hold. If $d_{\epsilon }(p,q) = d_{{\mathcal {T}}}(p,q)$ for all $p,q \in Y$, then these all hold since $d_{{\mathcal {T}}}$ restricted to *Y* is a metric. So, suppose this is not the case. To check that (A)–(C) hold, without loss of generality, by Lemma 2 we may assume that the convergence scenario $({\mathcal {T}},R,\epsilon )$ restricts to *Y* to give a convergence scenario $({\mathcal {Q}},R^*=\{r,r^*,s,s^*\},\epsilon )$ as in one of Fig. 3i–iv and that $d_{\epsilon }|_Y= d_{\epsilon }^{({\mathcal {Q}}, R^*)}$. We now check that (A)–(C) hold in each of the Cases (i)–(iv).

First, note that since $d_{\epsilon }$ is triplet respecting, we have

$$\begin{aligned} d_{\epsilon }(x,y) < \min \{d_{\epsilon }(x,z), d_{\epsilon }(y,z) \}, \end{aligned}$$

and so (A) must hold for all Cases (i)–(iv).

Moreover, in Cases (i) and (iv) $d_{\epsilon }(x,z) = d_{\epsilon }(y,z)$, and so in these cases (B) and (C) must always hold too. And, in Cases (ii) and (iii) $d_{\epsilon }(x,z) \le d_{\epsilon }(y,z)$, and so in these cases (B) holds. Hence, it suffices to show that (C) holds for Cases (ii) and (iii).

Let $\gamma$ be the height of $r^*$ and $s^*$ in the convergence scenario $({\mathcal {Q}},R^*=\{r,r^*,s,s^*\},\epsilon )$. Note that $\gamma \ge \alpha$. In Case (ii), (C) holds if and only if

$$\begin{aligned} d_{{\mathcal {T}}}(y,z)- 2 \epsilon (\beta - h(lca_T(x,y))) \le d_{{\mathcal {T}}}(y,x) +d_{{\mathcal {T}}}(x,z) - 2\epsilon (\beta - \gamma ). \end{aligned}$$

Since $d_{{\mathcal {T}}}(x,z)=d_{{\mathcal {T}}}(y,z)$ this last inequality holds if and only if

5 $$\begin{aligned} \epsilon \le \frac{d_{{\mathcal {T}}}(x,y)}{2(h(lca_T(x,y))-\gamma )}. \end{aligned}$$

But, since $\gamma \ge \alpha$, this last inequality holds by Inequality (4).

In Case (iii), (C) holds if and only if

$$\begin{aligned} d_{\epsilon }(y,z)=d_{{\mathcal {T}}}(y,z) \le d_{{\mathcal {T}}}(x,z) + d_{{\mathcal {T}}}(y,x) -2 \epsilon (\beta -\gamma ), \end{aligned}$$

which since $d_{{\mathcal {T}}}(x,z)=d_{{\mathcal {T}}}(y,z)$, holds if and only if

6 $$\begin{aligned} \epsilon \le \frac{d_{{\mathcal {T}}}(x,y)}{2(\beta - \gamma )}. \end{aligned}$$

Again, since $\gamma \ge \alpha$, this last inequality holds by Inequality (4).

($\Rightarrow$) Suppose that $d_{\epsilon }$ is a metric so that, in particular, (C) holds for every $x,y,z \in X$ distinct. Now, suppose $x,z \in X$ are distinct and strictly below *R* and $y \in X$ is such that $((x,y),z) \in {\mathcal {R}}(T)$ and $lca_T(x,y)$ is not below a bottom point of *R*. Then it follows that either Case (ii) or (iii) must hold in Fig. 3 and the height of $r^*$ and $s^*$ in the convergence scenario $({\mathcal {Q}},R^*=\{r,r^*,s,s^*\},\epsilon )$ given by restricting to $Y=\{x,y,z\}$ must be equal to $\alpha$. But, as shown above, (C) holds in Case (ii) if and only if Inequality (5) holds, from which Inequality (4) follows, and (C) holds in Case (iii) if and only if Inequality (6) holds, from which Inequality (4) again follows. $\square$

Note that since the right hand side of the inequality in the statement of Theorem 5 is always greater than 1, we have the following simple condition for ensuring that a triplet respecting distance is a metric.

### Corollary 6

Suppose $d_{\epsilon }$ is a triplet respecting distance. If $\epsilon \le 1$, then $d_{\epsilon }$ is a metric.

We now conclude this section by presenting a characterisation for when $d_{\epsilon }$ is a tree metric or an ultrametric in case it is a triplet respecting metric. We first state a useful lemma.

### Lemma 7

Suppose that $({\mathcal {T}}=(T,w),R,\epsilon )$ is a convergence scenario such that $d_{\epsilon }$ is a triplet respecting metric, and *T* is one of the trees in Figs. 4 or 5 with leaf set $\{x,y,z,t\}$. Then $d_{\epsilon }$ satisfies the 4-point condition if and only if *T* is one of the configurations in:

- Figure 4: (1), (2), (4), (7), (8), (9), (12), or
- Figure 5: (1), (2), (3), (7).

### Proof

Note that since $d_{\epsilon }$ is a metric we only need to check that the 4-point condition holds for *x*, *y*, *z*, *t* all pairwise distinct (i.e. we do not need to consider subsets of $\{x,y,z,t\}$). This is a straight-forward check using the fact that $d_{{\mathcal {T}}}$ satisfies the 4-point condition and Theorem 3. For example, the distance $d_{\epsilon }$ arising from tree *T* as in Fig. 5 configuration (6) satisfies $d_{\epsilon }(x,y) = d_{{\mathcal {T}}}(x,y)$, $d_{\epsilon }(t,z)= d_{{\mathcal {T}}}(t,z)$, $d_{\epsilon }(x,z)= d_{{\mathcal {T}}}(x,z)$, $d_{\epsilon }(y,t)= d_{{\mathcal {T}}}(y,t)$, $d_{\epsilon }(x,t) = d_{{\mathcal {T}}}(x,t)-2\epsilon (\beta -\alpha )$ and $d_{\epsilon }(y,z) = d_{{\mathcal {T}}}(y,z)$, and so, as $d_{{\mathcal {T}}}(x,y)+d_{{\mathcal {T}}}(t,z) < d_{{\mathcal {T}}}(x,z)+d_{{\mathcal {T}}}(y,t) = d_{{\mathcal {T}}}(x,t)+d_{{\mathcal {T}}}(y,z)$ (since $d_{{\mathcal {T}}}$ satisfies the 4-point condition),

$$\begin{aligned} \max \{d_{\epsilon }(x,y)+d_{\epsilon }(t,z), d_{\epsilon }(x,z) + d_{\epsilon }(y,t) \} < d_{\epsilon }(x,t) + d_{\epsilon }(y,z), \end{aligned}$$

from which it follows that $d_{\epsilon }$ does not satisfy the 4-point condition. However, in Fig. 5 configuration (1), we have $d_{\epsilon }(x,y) = d_{{\mathcal {T}}}(x,y)$, $d_{\epsilon }(z,t)= d_{{\mathcal {T}}}(z,t)$, $d_{\epsilon }(x,z)= d_{{\mathcal {T}}}(x,z)-2\epsilon (\beta -\alpha )$, $d_{\epsilon }(y,t)= d_{{\mathcal {T}}}(y,t)-2\epsilon (\beta -\alpha )$, $d_{\epsilon }(x,t) = d_{{\mathcal {T}}}(x,t)-2\epsilon (\beta -\alpha )$ and $d_{\epsilon }(y,z) = d_{{\mathcal {T}}}(y,z)-2\epsilon (\beta -\alpha )$. Therefore, $d_{\epsilon }$ satisfies the 4-point condition since $d_{{\mathcal {T}}}(x,y)+d_{{\mathcal {T}}}(z,t) < d_{{\mathcal {T}}}(x,z)+d_{{\mathcal {T}}}(y,t) = d_{{\mathcal {T}}}(x,t)+d_{{\mathcal {T}}}(y,z)$ (since $d_{{\mathcal {T}}}$ satisfies the 4-point condition) and by Theorem 3$d_{{\mathcal {T}}}(x,z) - d_{{\mathcal {T}}}(x,y) > 2\epsilon (\beta -\alpha )$ and $d_{{\mathcal {T}}}(y,t) - d_{{\mathcal {T}}}(z,t) > 2\epsilon (\beta -\alpha )$ both hold, from which it follows that

$$\begin{aligned} d_{\epsilon }(x,y)+ d_{\epsilon }(z,t) < d_{\epsilon }(x,z)+d_{\epsilon }(y,t) = d_{\epsilon }(x,t) + d_{\epsilon }(y,z). \end{aligned}$$

$$\square $$

**Fig. 6:**
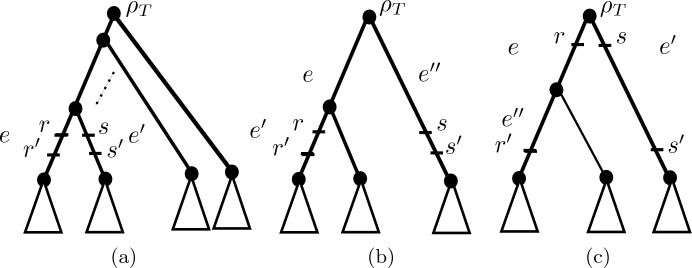
Possible placements of $r,r',s,s'$ for the proof of Theorem 8

### Theorem 8

Suppose that $({\mathcal {T}}=(T,w),R,\epsilon )$ is a convergence scenario such that $d_{\epsilon }$ is a triplet respecting metric. Then $d_{\epsilon }$ is a tree metric if and only if precisely one of the following holds (see Fig. 6):

- There exist edges $e,e'$ in *T* such that $|e \cap e'| =1$ and $|R \cap e|=|R \cap e'|=2$.
- There exist edges $e,e',e''$ in *T* such that *e* and $e''$ both contain the root of *T*, $|e \cap e'| =1$, $|e' \cap e''|= 0$, and $|R \cap e'|=|R \cap e''|=2$.
- There exist edges $e,e',e''$ in *T* such that *e* and $e'$ both contain the root of *T*, $|e \cap e''|= 1$, $|R \cap e|=|R \cap e''|=1$ and $|R \cap e'|=2$.

Moreover, $d_{\epsilon }$ is an ultrametric if and only if (a) holds. In particular, if $d_{\epsilon }$ is a triplet respecting tree metric (or ultrametric), then $d_{\epsilon }=d_{(T,w')}$ where $w'$ is some (necessarily unique) edge weighting of *T*.

### Proof

We begin by considering the first statement of the theorem. If $|X|=3$ then the statement holds since any metric on a set of size 3 is a tree metric, and precisely one of the cases (a)-(c) can apply since they detail all possible convergence scenarios on *X*. So assume for the remainder of the proof that $|X|\ge 4$.

Now, suppose $d_{\epsilon }$ is a tree metric on *X*. Put $R=\{r,r',s,s'\}$ and $\rho =\rho _T$. To see that one of (a)–(c) holds, we perform a case analysis in which we show that one of (a)–(c) must hold or that we can find a subset $Y\subseteq X$ of *X* of size 4 so that, in view of Lemma 7, $d_{\epsilon }|_Y$ is not a tree metric which is impossible.

Suppose first that neither of the top points in *R* is contained in an edge of *T* that contains $\rho$. We claim that (a) must hold. To see why this is the case, let *e* be an edge of *T* that contains *r*. Then $lca(r,s) \ne \rho$ since otherwise we can find leaves $x,y,z,t \in X$ such that the convergence scenario obtained by restricting *T* to $Y=\{x,y,z,t\}$ would be as in Fig. 5(6), which is impossible by Lemma 7. Moreover, *r* and $r'$ must both be contained in *e* since otherwise we could choose elements $x,y,z,t\in X$ such that *T* restricted to $Y=\{x,y,z,t\}$ would be as in Fig. 4(10) which is impossible. Similarly, as $r,r'$ are both in *e*, *s* and $s'$ must both be contained in the same edge $e'$ of *T* since otherwise we could obtain a contradiction using Fig. 4(10) again by reversing the roles of $r,r'$ and $s,s'$. And, finally, $e \cap e' \ne \emptyset$ since otherwise (reversing the roles of $r,r'$ and $s,s'$ if necessary), we could choose elements $x,y,z,t\in X$ such that *T* restricted to $Y=\{x,y,z,t\}$ would be as in Fig. 4(11) which is impossible. So (a) must hold as claimed.

Now, assume *r* is in edge *e* of *T* with $\rho \in e$ so that, in particular, $lca(R)=\rho$. Then either $r'$ is contained in *e* or $r'$ is contained in an edge $e''$ with $|e''\cap e|=1$. Indeed, if this were not the case, then there would exist at least two vertices in *V*(*T*) that are contained in the path in *T* between *r* and $r'$. So, we could find leaves $x,y,z,t \in X$ such that the convergence scenario obtained by restricting *T* to $Y=\{x,y,z,t\}$ would be as in Fig. 4(3) which is impossible.

Let $e'$ be the edge in *T* that contains *s*. We first consider the case that $r'$ is contained in *e*. Note that in the case $\rho \in e'$. Indeed, if not, then (b) must hold since otherwise we could find leaves $x,y,z,t \in X$ such that the convergence scenario obtained by restricting *T* to $Y=\{x,y,z,t\}$ would be as in Fig. 4(5) or (6) with the roles of $r,r'$ and $s,s'$, reversed, which is impossible. Note also that $s'$ must either be in $e'$ or in an edge $e''$ of *T* with $e' \cap e'' \ne \emptyset$, otherwise we could obtain a configuration as in Fig. 4(3) with the roles of $r,r'$ and $s,s'$ reversed. But then (a) holds if $s'$ is contained in $e'$ and otherwise (b) holds.

Finally, suppose $r'$ is not contained in *e*. Then $\rho \in e'$ since otherwise we can find $x,y,z,t \in X$ and use Fig. 5(5) to obtain a contradiction. Moreover, $s'$ is contained in $e'$ otherwise we could find $x,y,z,t \in X$ and use Fig. 5(4) to obtain a contradiction. Thus (c) must hold.

Conversely, suppose precisely one of (a)–(c) holds. We will show that $d_{\epsilon }$ is a tree metric. Take any $Y \subseteq X$ with $|Y|=4$. It suffices to show that $d_{\epsilon }|_Y$ is a tree metric. If $d_{\epsilon }|_Y=d_{{\mathcal {T}}}|_Y$ then this is clearly the case. So we may assume $d_{\epsilon }|_Y \ne d_{{\mathcal {T}}}|_Y$.

If one of (a) or (b) holds, then since $d_{\epsilon }|_Y \ne d_{{\mathcal {T}}}|_Y$ there must be some pair of elements in *Y* that is strictly below *R*. It follows that we can assume that $Y=\{x,y,z,t\}$ and that ${\mathcal {T}}$ restricted to *Y* must be as in Figs. 4(1),(4),(8), (9), (12) or 5(1),(3),(7). In either of these cases $d_{\epsilon }|_Y$ must be a tree metric by Lemma 7.

Similarly, if (c) holds then since $d_{\epsilon }|_Y \ne d_{{\mathcal {T}}}|_Y$ there must be an element in *Y* that is below $s'$. Also, there must be (i) an element in *Y* that is below *r* but not below $r'$ or (ii) an element in *Y* that is below $r'$. If both (i) and (ii) hold, it follows that we can assume that $Y=\{x,y,z,t\}$ and that ${\mathcal {T}}$ restricted to *Y* must be as in Figs. 5(2) or 4(2),(7). In either of these cases $d_{\epsilon }|_Y$ must be a tree metric by Lemma 7. If only one of (i) or (ii) holds, then a similar argument can be used where we may need to restrict to four elements in *X* to obtain a convergence scenario in a similar way to that used in Lemma 2 before applying Lemma 7.

We now consider the second statement in the theorem. First, suppose that (a) holds so that there exist edges $e,e'$ in *T* such that $r,r'$ are points in *e*, $s,s'$ are points in $e'$ and $|e \cap e'| =1$. To see that $d_{\epsilon }$ is an ultrametric, we need to show that Inequality (2) holds for all $x,y,z \in X$ distinct, i.e. that two of the values $d_{\epsilon }(x,y)$, $d_{\epsilon }(x,z)$ and $d_{\epsilon }(y,z)$ are equal and not less than the third. It clearly suffices to show that this is the case for $x \in X$ below $r'$ in *T*, $y \in X$ below $s'$ in *T*, and $z \in X$.

If ((*x*, *y*), *z*) is a triplet in ${\mathcal {R}}(T)$, then it easily seen that $d_{\epsilon }(x,y) < d_{\epsilon }(x,z) = d_{\epsilon }(y,z)$. Otherwise, we can assume without loss of generality that ((*x*, *z*), *y*) is a triplet in ${\mathcal {R}}(T)$. Since $d_{\epsilon }$ is triplet respecting, $d_{\epsilon }(x,z) \le \min \{d_{\epsilon }(x,y), d_{\epsilon }(z,y)\}$ follows. Clearly $d_{\epsilon }(x,y)=d_{\epsilon }(z,y)$ and so $d_{\epsilon }(x,z) \le d_{\epsilon }(x,y) = d_{\epsilon }(y,z)$.

Conversely, assume that $d_{\epsilon }$ is an ultrametric. Then $d_{\epsilon }$ is a tree metric. Hence, precisely one of (a)–(c) in Theorem 8 must hold. We now show that neither (b) nor (c) can hold, which will complete the proof of the theorem.

So, assume for contradiction that either (b) or (c) holds. If (b) holds, then pick $x \in X$ below $r'$ in *T*, $z\in X$ below $s'$ in *T* and $y\in X$ below the vertex in $e \cap e'$ but not below *r* in *T*. And, if (c) holds, then pick $x \in X$ below $r'$ in *T*, $z\in X$ below $s'$ in *T* and $y\in X$ below *r* but not below $r'$ in *T*. In either case, since $d_{\epsilon }$ is a triplet respecting ultrametric we must have $d_{\epsilon }(y,z)=d_{\epsilon }(x,z)$ (as the two largest values of $d_{\epsilon }|_{\{x,y,z\}}$ must be equal). But this is clearly impossible since, by the definition of $d_{\epsilon }$, we must have $d_{\epsilon }(y,z) >d_{\epsilon }(x,z)$ in both cases (b) and (c).

The last statement of the theorem holds in view of (Semple and Steel, 2003, Theorems 7.1.8 and 7.2.5). $\square$

### Remark 1

There are examples where $d_{\epsilon }$ is an ultrametric but the characterisation given in Theorem 8 does not hold. For example, take Fig. 4(7) with $h(\rho _T)=101$, $h(lca_{T}(x,z))=1$, $h(lca_{T}(x,y))=98\frac{1}{2}$, $\alpha =98$, $\beta =99$, and $\epsilon =5$. However, note that in this example $d_{\epsilon }$ is not triplet respecting.

## Recovering the height of the tree within a convergence scenario

Consider the two convergence scenarios given in Fig. 4(7) with $h(\rho _T)=5$, $h(lca(x,y))=3$, $h(lca(x,z))=1$, $\alpha =2\frac{1}{2}$ and $\beta =3\frac{1}{2}$ and in Fig. 4(8) with $h(\rho _T)=4\frac{1}{2}$, $h(lca(x,y))=3$, $h(lca(x,z))=1$, $\alpha =1\frac{1}{2}$ and $\beta =2$. Then it can be checked that, for $\epsilon =1$, both scenarios give rise to the same map $d_{\epsilon }$ which, in view of Lemma 1 is a distance, in view of Theorem 3 is triplet respecting and, in view of Theorem 5 is a metric even though the height of ${\mathcal {T}}$ in the two scenarios is different. In particular, for this example, even though we can recover the topology of ${\mathcal {T}}$ from $d_{\epsilon }$ since it is a triplet respecting metric, we are not able to identify the height of ${\mathcal {T}}$ from $d_{\epsilon }$ in the sense that there are different choices of *R* (but with the same $\epsilon$ and *T*) which induce the same $d_{\epsilon }$. Motivated by this example, in this section for a convergence scenario $({\mathcal {T}},R,\epsilon )$ that gives rise to a triplet respecting metric, we shall characterise which choices of *R* ensure that we are able to recover the height of ${\mathcal {T}}$ from $d_{\epsilon }$ (see Theorem 11 below).

To make this more precise, for a convergence scenario $({\mathcal {T}}=(T,w),R,\epsilon )$ we denote $d_{\epsilon }$ also by $d^{(w,R)}_{\epsilon }$ to emphasise the choice of *w* and *R*. In addition, for some choice of *w* and *R*, we shall say that the height of (*T*, *w*) is *identifiable* from $d^{(w,R)}_{\epsilon }$ if there does not exist a choice of $w'$ and $R'$ with $(w',R') \ne (w,R)$ such that $d^{(w,R)}_{\epsilon } = d^{(w',R')}_{\epsilon }$ and $h_{(T,w)}(\rho _T) \ne h_{(T,w')}(\rho _T)$.

We now give some key examples of some choices of *R* in a convergence scenario where it is not possible to identify the height of the underlying tree. From now on, given a convergence scenario $({\mathcal {T}}=(T,w),R,\epsilon )$, we put $h_w(a)=h_{(T,w)}(a)$ for any point *a* in ${\mathcal {T}}$ if *T* is clear from the context. Note that in the following lemma we only require that $d_{\epsilon }$ is a triplet respecting distance.

### Lemma 9

**Fig. 7:**
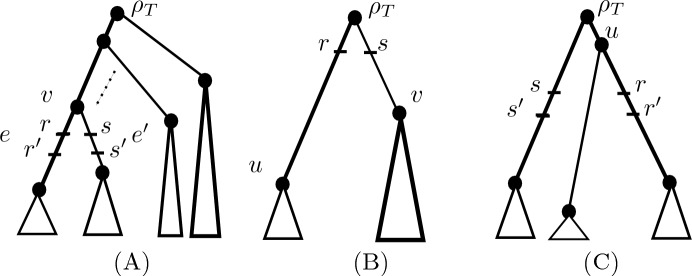
The convergence scenarios considered in Theorem 11. The edge-weights assigned by *w* to *T* are arbitrary, but chosen so that $d_{\epsilon }^{(w,R)}$ is a triplet respecting metric. In **B**, $r'$ and $s'$ can be in any edge of *T* below *r* and *s*, respectively. Note that the roles of $r,r'$ and $s, s'$ are interchangeable

Suppose that $({\mathcal {T}},R,\epsilon )$ is a convergence scenario such that $d_{\epsilon }$ is a triplet respecting distance. If *R* is one of the configurations in Fig. 7, then the height of ${\mathcal {T}}$ is not identifiable from $d_{\epsilon }$.

### Proof

Put ${\mathcal {T}}=(T,w)$ and $\rho =\rho _T$. For *R* as in Fig. 7, let $l=h_w(r)-h_w(r')=h_w(s)-h_w(s')$. We now consider each of the configurations (A)–(C) in Fig. 7.

First, suppose $R=\{r,r',s,s'\}$ is as in Fig. 7A. Let $v \ne \rho$ denote the vertex in this configuration that is contained in the edges *e*, $e'$ which contain $r,r',s,s'$. Note that for any leaf *x* below *e* and any leaf *y* below $e'$, we have $d_{\epsilon }(x,y) = 2h_w(v) - 2\epsilon l$.

We first claim that $\min \{w(e),w(e')\} > \epsilon l$. Indeed, suppose that there are precisely two leaves *x* and *y* below *v*. Then *x* and *y* form a cherry of *T*. Hence, $w(e)=w(e')$ because (*T*, *w*) is equidistant. Since $d^{(w,R)}_{\epsilon }$ is a distance

$$\begin{aligned} d^{(w,R)}_{\epsilon }(x,y)=d_{{\mathcal {T}}}(x,y) - 2 \epsilon l = 2w(e) - 2 \epsilon l >0, \end{aligned}$$

and so $w(e) > \epsilon l$. If there are at least three leaves in *T* below *v*, then we may assume without loss of generality that $w(e)=\min \{w(e),w(e')\}$, so that in particular there must be at least two leaves below $r'$. Let *x* and *y* be two leaves below $r'$ such that $lca_T(x,y)$ is a child of *v*, and let *z* be a leaf below $s'$. Then $d_{{\mathcal {T}}}(x,z) - d_{{\mathcal {T}}}(x,y) = 2w(e)$. Since $d^{(w,R)}_{\epsilon }$ is triplet respecting but not a cherry scenario it follows by Theorem 3 that $w(e) > \epsilon l$. So the claim follows.

Now, consider the edge-weighting $w'$ of *T* that is obtained as follows. Add $\epsilon \delta$, some $\delta >0$ small, to the weights of the two edges containing the root of *T*, subtract $\epsilon l$ from the weights of the edges *e* and $e'$ (which is possible since $\min \{w(e),w(e')\} > \epsilon l$), add $\epsilon l$ to the weight of the edge containing *v* and not equal to $e, e'$ (which exists as $v \ne \rho$), and keep all other edge weights the same. In addition, place $p,p'$ into one edge containing $\rho$ and $q,q'$ into the other edge containing the root of *T* so that $h_{w'}(p)-h_{w'}(p')=h_{w'}(q)-h_{w'}(q')=\delta$, which is possible by taking $\delta$ to be sufficiently small. Then for $R' = \{p,p',q,q'\}$, it is straight-forward to check that $(w,R)\ne (w',R')$, $d^{(w,R)}_{\epsilon } = d^{(w',R')}_{\epsilon }$, and $h_w(\rho ) \ne h_{w'}(\rho )$ (since $h_{w'}(\rho ) = h_w(\rho ) + \epsilon \delta$).

Now, suppose $R=\{r,r',s,s'\}$ is as in Fig. 7B where $r'$ and $s'$ are as indicated in its caption. Consider the edge-weighting $w'$ of the tree *T* obtained by replacing the weights of the edges in *T* containing $\rho$ with the same weight plus $\epsilon \delta$ for some small $\delta >0$, and keeping all other edges the same weight. Relative to the weighting $w'$, place *p*, *q* in the same edges of *T* as *r*, *s*, respectively, at height $h_{w}(r)+\delta$, $h_{w}(s)+ \delta$, respectively (which is possible since we can choose $\delta$ to be sufficiently small), and place $p',q'$ in the same edges as $r',s'$ with heights (relative to $w'$) $h_{w}(r')$ and $h_{w}(s')$, respectively. Then for $R' = \{p,p',q,q'\}$, it is straight-forward to check that $(w,R)\ne (w',R')$, $d^{(w,R)}_{\epsilon } = d^{(w',R')}_{\epsilon }$ and $h_w(\rho ) \ne h_{w'}(\rho )$ (since $h_{w'}(\rho ) = h_w(\rho ) + el$).

Finally, suppose $$R=\{r,r',s,s'\}$$ is as in Fig. 7C. Consider the weighting $$w'$$ of *T* that is obtained by replacing the weights of the edges in *T* containing $$\rho $$ with the same weight plus $$\epsilon \delta $$, some $$\delta >0$$ small, and keeping all other edges the same weight. Let *u* be the vertex in *T* adjacent to $$\rho $$ and above $$r,r'$$. Relative to the weighting $$w'$$, place *q* at height $$h_{w}(u)+\delta $$ and *p* at the same height in the other edge that contains the root of *T* (which is possible by taking $$\delta $$ sufficiently small). Also, place $$q'$$ in the same edge as $$r'$$ at height $$h_{w}(u)-l $$ (which is possible since the edge containing $$r,r'$$ in *T* with weight *w* has length greater than *l*), and $$p'$$ at the same height in the same edge that contains *p*. Then for $$R' = \{p,p',q,q'\}$$, it is straight-forward to check that $$(w,R)\ne (w',R')$$, $$d^{(w,R)}_{\epsilon } = d^{(w',R')}_{\epsilon }$$, and $$h_w(\rho ) \ne h_{w'}(\rho )$$ (since $$h_{w'}(\rho ) = h_w(\rho ) + \epsilon \delta $$). $$\square $$

We now prove a technical lemma that we will use to prove the main result of this section. Note that this result does not depend on edge-weights.

### Lemma 10

Suppose that $({\mathcal {T}}=(T,w),R,\epsilon )$ is a convergence scenario. Then *R* is *not* as in one of the configurations pictured in Fig. 7B or C if and only if precisely one of the Conditions (a)–(e) below holds:

- There are $x,y,z,t \in X$ such that $lca_T(x,t)=\rho _T$, $u=lca_T(x,y)$ and $v=lca_T(z,t)$ are the children of $\rho _T$, one top point in *R* is in the edge $\{\rho _T,v\}$, one bottom point of *R* lies on the path between *v* and *t* and two points in *R* lie on the path between *u* and *x*.
- There are $x,y,z,t \in X$ such that $lca_T(x,t)=\rho _T$, two points in *R* lie on the path between $\rho _T$ and *x*, $u=lca_T(y,t)$ is a child of $\rho _T$, $v=lca_T(z,t)$ is a child of *u*, and two points in *R* lie on the path between *v* and *t*.
- There are $x,y,z,t \in X$ such that $lca_T(x,t)=\rho _T$, two points in *R* lie on the path between $\rho _T$ and *x*, $u=lca_T(y,t)$ is a child of $\rho _T$, $v=lca_T(z,t)$ is a child of *u*, one top point in *R* is in the edge $\{u,v\}$, and one bottom point in *R* lies on the path between *v* and *t*.
- There are $x,y,z,t \in X$ such that $u=lca_T(x,y)$ and $v=lca_T(z,t)$ are the children of $\rho _T$, two points in *R* lie on the path in *T* between *u* and *x*, and two points in *R* lie on the path in *T* between *v* and *z*.
- All of the points in *R* are contained in one of the subtrees of *T* whose root is a child of $\rho _T$.

### Proof

It is straight-forward to see that if any of Conditions (a)–(e) holds then *R* is *not* as in Fig. 7B or C.

Conversely, suppose that *R* is *not* as in Fig. 7B or C. First note that we may assume that $lca(R)=\rho _T$, otherwise (e) holds.

Suppose first that one of the top points in *R*, say *r*, is in an edge of *T* that contains the root $\rho _T$ of *T*. By Fig. 7B, the top point *s* is not contained in the other edge incident with $\rho _T$. Now, if the point $r' \in R$ below *r* is not contained in the same edge as *r*, then (a) holds. On the other hand, if $r'$ is contained in the same edge as *r*, then as Fig. 7C cannot hold it follows that (c) holds in case *s* is in an edge incident with a child of $\rho _T$, and that (b) holds otherwise.

Now, suppose that *r* is in an edge of *T* that does not contain the root of *T* and $s\in R$ is the other top point. By the preceding paragraph with the role of *s* and *r* interchanged, we can assume that *s* is also in an edge of *T* that does not contain the root of *T*. But then (d) holds. $\square$

We now prove the main result of this section.

### Theorem 11

Suppose that $({\mathcal {T}}=(T,w),R,\epsilon )$ is a convergence scenario such that $d_{\epsilon } = d^{({\mathcal {T}},R)}_{\epsilon }$ is a triplet respecting metric. Then the height of ${\mathcal {T}}$ is identifiable from $d_{\epsilon }$ if and only if *R* is *not* as one of the configurations in Fig. 7. Moreover, if this is the case, then $h_{{\mathcal {T}}}(\rho _T)=\frac{1}{2}\max _{x,y \in X}\{d_{\epsilon }(x,y)\}$.

### Proof

The ‘if’ direction follows immediately by Lemma 9.

For the ‘only if direction’, let $h=h_w$, $h'=h_{w'}$ and, for *u* a point in ${\mathcal {T}}$ or $(T,w')$, let $h(u)=h_w(u)$ or $h'(u)=h_{w'}(u)$, respectively. Furthermore, put $\rho =\rho _T$, $h'=h'(\rho )$ and $h=h(\rho )$. To see that this direction holds, suppose that $R=\{r,r',s,s'\}$ is *not* as in Fig. 7A–C and assume for contradiction that the height of ${\mathcal {T}}$ in not identifiable from $d_{\epsilon }$. Then there exists some $(w',R'=\{p,p',q,q'\})\ne (w,R)$ with $h'\not =h$. such that $d^{(w,R)}_{\epsilon }= d^{(w',R')}_{\epsilon }$.

First note that since *R* is *not* as in Fig. 7A–C, and, in particular, not as in Fig. 7B, C, we may assume that one of Lemma 10(a)–(e) holds and that if Lemma 10(e) holds then *R* is not as in Fig. 7A. Using the notation in Lemma 10(a)–(e), we now show that each of these cases leads to a contradiction, which will complete the proof of the first part of the theorem.

First note that if (a) holds, then since $d^{(w',R')}_{\epsilon }(y,z)=d^{(w,R)}_{\epsilon }(y,z)=2h$, and $h' \ne h$, it follows that $h'>h$, and that *p*, *q* are contained in the two edges that contain the root of *T*. A similar argument can also be applied to each of the cases (b)–(e) to show that in all of these cases *p*, *q* must be in the two edges that contain the root of *T*, so we shall assume this from now on.

**Lemma** 10**(a) holds:** Without loss of generality assume that *r* and $r'$ are on the path from *u* to *x*, *s* is in the edge $\{\rho ,v\}$, and $s'$ is on the path from *v* to *t* in *T*. Note that this implies $h(u)>h(v)$. Also, without loss of generality, we assume that *p* is above *u* and *q* is above *v* in $(T,w')$.

Now, $lca(q',t) \ne v$ since $d^{(w',R')}_{\epsilon }(x,z)=d^{(w,R)}_{\epsilon }(x,z) > d^{(w,R)}_{\epsilon }(x,t)=d^{(w',R')}_{\epsilon }(x,t)$, and $lca_T(p',x) \ne u$ since $d^{(w',R')}_{\epsilon }(z,y)=d^{(w,R)}_{\epsilon }(z,y) > d^{(w,R)}_{\epsilon }(z,x)=d^{(w',R')}_{\epsilon }(z,x)$. Moreover, $q'$ is not above *v* since otherwise $d^{(w',R')}_{\epsilon }(x,z)=d^{(w,R)}_{\epsilon }(x,z) > d^{(w,R)}_{\epsilon }(x,t)=d^{(w',R')}_{\epsilon }(x,t)$ which is impossible. Hence, using a similar argument, we see that $q'$ must in fact lie on the path from *v* to *t* as $d^{(w,R)}_{\epsilon }(b,x) \ge d^{(w,R)}_{\epsilon }(b,t)$ for all $b \ne t$ with $lca_T(b,z)=v$. Note also that $p'$ is not above *u* since otherwise $h(v)=h'(v)>h'(u)=h(u)$ which is a contradiction. In addition, since $d^{(w,R)}_{\epsilon }(b,t) \ge d^{(w,R)}_{\epsilon }(t,x)$ for all $b \ne x$ with $lca_T(b,y)=u$, it follows that $p'$ must lie on the path from *u* to *x*.

Now, suppose $h'=h+\gamma$ ($\gamma >0$), $\delta =h'(p)-h'(u)$, $\tau =h'(u)-h'(p')$ and $\kappa =h(s)-h(v)$. Then, since $h'(u)=h(u)>h(v)=h'(v)$, $d^{(w,R)}_{\epsilon }(y,z)=2\,h=d^{(w',R')}_{\epsilon }(y,z)=2(h+\gamma )-2\epsilon \delta$. So $\gamma = \epsilon \delta$. Also, $d^{(w,R)}_{\epsilon }(x,z)=2\,h-2\epsilon \kappa = d^{(w',R')}_{\epsilon }(x,z)= 2(h+\epsilon \delta ) -2 \epsilon (\delta + \tau )$. Hence $\kappa = \tau$. But this is impossible, since $\kappa < h(u)-h(v)$ and $\tau >h(u)-h(v)$.

**Lemma** 10**(b) holds:** Without loss of generality assume that $r,r'$ are on the path from $\rho$ to *x* and $s,s'$ are on the path from *v* to *t*. Also, without loss of generality, assume that *p* is on the path from $\rho$ to *x* and *q* is in the edge $\{\rho ,u\}$ in $(T,w')$.

Note that $q'$ cannot be in the same edge $\{\rho ,u\}$ as *q* since this implies $d^{(w',R')}_{\epsilon }(x,y)=d^{(w,R)}_{\epsilon }(x,y)> d^{(w,R)}_{\epsilon }(x,t)=d^{(w',R')}_{\epsilon }(x,t)$ which is impossible. Thus $q'$ must be below *u*. But then we would have $d^{(w',R')}_{\epsilon }(x,y)=d^{(w,R)}_{\epsilon }(x,y)= d^{(w,R)}_{\epsilon }(x,z)=d^{(w',R')}_{\epsilon }(x,z)$ which is also impossible.

**Lemma** 10**(c) holds:** Without loss of generality, assume that $r,r'$ are on the path from $\rho$ to *x*, *s* is in the edge $\{u,v\}$ and $s'$ is on the path from *v* to *t* in $(T,w')$. Without loss of generality, we assume that *q* is contained in the edge $\{\rho ,u\}$.

Using similar arguments to the ones in case (a), note that $lca_T(q',y) \ne v$ since $d^{(w,R)}_{\epsilon }(y,x) > d^{(w,R)}_{\epsilon }(z,x)$, $q'$ is not in the edge $\{u,v\}$ and $lca_T(q',t)\ne v$ since $d^{(w,R)}_{\epsilon }(z,x) > d^{(w,R)}_{\epsilon }(t,x)$, and thus $q'$ is on the path from *v* to *t* since $d^{(w,R)}_{\epsilon }(b,x) \ge d^{(w,R)}_{\epsilon }(b,t)$ for all $b \ne t$ with $lca_T(b,z)=v$.

So, suppose $h'=h+\gamma$ ($\gamma >0$), $\kappa =h(s)-h(v)$, $\tau =h'(u)-h'(v)$ and $\delta =h'(q)-h'(u)$. Then $2\,h=d^{(w,R)}_{\epsilon }(x,y)=d^{(w',R')}_{\epsilon }(x,y)=2(h+\gamma )-2\epsilon \delta$. So $\gamma = \epsilon \delta$. Also, $2\,h-2\epsilon \kappa = d^{(w,R)}_{\epsilon }(x,z)= d^{(w',R')}_{\epsilon }(x,z)= 2(h+\epsilon \delta ) -2 \epsilon (\delta + \tau )$. Hence $\kappa =\tau$. But this is impossible since $\kappa <h(u)-h(v) =h'(u)-h'(v) = \tau$.

**Lemma** 10**(d) holds:** Without loss of generality, assume $r,r'$ lie on the path in *T* between *u* and *x*, and that $s,s'$ lie on the path in *T* between *v* and *z*. Without loss of generality, we assume that *p* is contained in the edge $\{\rho ,u\}$.

Note that as $d^{(w,R)}_{\epsilon }$ is a triplet respecting metric, by Theorem 8$d^{(w,R)}_{\epsilon }$ is not an ultrametric, and so $p'$ and $q'$ are not both in the same edges as *p* and *q*, respectively (otherwise, by Theorem 8, $d_{\epsilon }$ would be an ultrametric).

Without loss of generality, suppose that $p'$ is not in the same edge as *p*. Then $p'$ must be on the path in $(T,w')$ from *u* to *x*, otherwise there would be some $b \in X-\{x\}$ below $p'$ with $lca_T(b,x)=u$ or $lca_T(b,x)$ below *u*, which implies $d^{(w',R')}_{\epsilon }(x,z) > d^{(w',R')}_{\epsilon }(b,z)$, which contradicts $d^{(w,R)}_{\epsilon }(x,z) = d^{(w,R)}_{\epsilon }(b,z)$. A similar argument also implies that $q'$ must be on the path in *T* from *q* to *z*.

Now if $q'$ is below *v*, then this leads immediately to a contradiction since it implies that $d^{(w',R')}_{\epsilon }(y,z) < d^{(w',R')}_{\epsilon }(y,t)$, but $d^{(w,R)}_{\epsilon }(y,z) = d^{(w,R)}_{\epsilon }(y,t)$. And if $q'$ is in the same edge $\{\rho ,v\}$ as *q*, then we again obtain a contradiction since then $d^{(w',R')}_{\epsilon }(x,z) = d^{(w',R')}_{\epsilon }(x,t)$, but $d^{(w,R)}_{\epsilon }(x,z) < d^{(w,R)}_{\epsilon }(x,t)$.

**Lemma** 10**(e) holds and ***R* **is not as in Fig.** 7**A:** Note that as Lemma 10(e) holds, we must have $d^{(w,R)}_{\epsilon }(x,y)=2h=d^{(w',R')}_{\epsilon }(x,y)$ for all *x*, *y* in *X* with $lca_T(x,y)=\rho _T$. But then, as $h \ne h'$, it is straight-forward to check that *p* and $p'$ must be in the same edge of *T* that contains the root, and that the same holds for *q* and $q'$. Hence, since $d_{\epsilon }$ is a triplet respecting metric by assumption it follows that $d^{(w',R')}_{\epsilon }$ is an ultrametric by Theorem 8. But this is impossible since *R* is not as in Fig. 7A and so, by Theorem 8, $d^{(w,R)}_{\epsilon }=d^{(w',R')}_{\epsilon }$ is not an ultrametric.

The last statement of the theorem holds since if *R* is one of the configurations in Lemma 10, then it is straight-forward to check that there must exist some $a,b\in X$ such that $d^{(w,R)}_{\epsilon }(a,b)=2h$ (since in all of (a)–(e) at least one of the edges in *T* containing the root of *T* does not contain a top point of *R*), and clearly $d^{(w,R)}_{\epsilon }(x,y) \le 2h$ for all $x,y \in X$. $\square$

### Corollary 12

Suppose that $({\mathcal {T}},R,\epsilon )$ is a convergence scenario such that $d_{\epsilon }$ is a triplet respecting tree metric. Then the height of ${{\mathcal {T}}}$ is *not* identifiable from $d_{\epsilon }$.

### Proof

If $d_{\epsilon }$ is a tree metric, then, by Theorem 8, *R* must be as in Fig. 6. But if the height of ${\mathcal {T}}$ is identifiable from $d_{\epsilon }$, then, by Theorem 11, either Lemma 10(a)–(d) holds or Lemma 10(e) holds and *R* is not as in Fig. 7A. But it is straight-forward to check that this is impossible. $\square$

### Remark 2

The situation in Theorem 11 gets more complicated if we allow the $\epsilon$ parameter to also vary. For example, consider the convergence scenario $({\mathcal {T}}=(T,w),R,\epsilon )$ in Fig. 5(5) where the weight of the edge $\{\rho _T, lca_{T}(x,y)\}$ is 2, $h(lca_{T}(x,y))=4$, $h(lca_{T}(z,t))=2$, $\beta =3$, $\alpha =\frac{3}{2}$ and $\epsilon =\frac{1}{2}$, and the convergence scenario $({\mathcal {T}}=(T,w'),R',\epsilon ')$ in Fig. 5(4) where the weight of the edge $\{\rho _T, lca_{T}(x,y)\}$ is $2\,\frac{1}{4}$, $h(lca_{T}(x,y))=4$, $h(lca_{T}(z,t))=2$, $\beta '=5$ and $\alpha '=1$, where $\epsilon '=\frac{1}{4}$. Then $d_{\epsilon }^{(w,R)}$ is a triplet respecting metric. Moreover, $d_{\epsilon '}^{(w',R')}=d_{\epsilon }^{(w,R)}$ and $h_{{\mathcal {T}}}(\rho _T)=6\,\frac{1}{4} \ne 6= h_{{\mathcal {T}}'}(\rho _T)$, even though the configuration of *R* in *T* is not as in Fig. 7 (since *R* corresponds to the configuration given in Lemma 10(a)). It could be interesting to understand when the height of ${\mathcal {T}}$ is identifiable in case the $\epsilon$ parameter is also allowed to vary.

## Discussion

We have introduced a new distance-based model for convergent evolution and characterised when the model leads to a tree metric, as well as giving conditions in terms of the model’s parameters for when it still possible to recover the underlying tree and its height even in case we do not obtain a tree metric. Our model is similar in nature to the convergence-divergence models presented in Mitchell et al. (2018) in which a probabilistic approach is developed based on a Markov model of character evolution. In our distance-based approach convergence is acting in a linear way, whereas in the character-based approach two sequences that are converging converge faster when they are further apart and more slowly as they get closer since there are fewer mismatch sites to “correct”.

In Mitchell et al. (2018), the authors mainly focus on phylogenetic trees that have three or four leaves, where they also find cases in which convergence gives rise to tree metrics (e.g. in (Mitchell et al., 2018, Fig. 6) they give an example similar to our Fig. 4(8)). Since our model can be applied to a set of species of arbitrary size it could be interesting to understand if there are deeper connections between the two approaches that could be exploited to give further insights into convergent evolution for larger data sets. A starting point might be to consider the interplay of our approach with the Jukes-Cantor model (Jukes and Cantor 1969), one of the simplest Markov models that is used to correct distance data in evolutionary studies [see e.g. (Felsenstein, 2004, Chapter 11)].

Our results suggest that under some circumstances the tree topology and some information about convergence events may be recoverable from observed distances. We give conditions for recovering the topology of the underlying tree and its overall height. In general, the starting and ending points $\alpha$ and $\beta$ are not precisely recoverable as they only effect the distances via their difference $\beta - \alpha$. However, if $\epsilon$ is assumed known it may be possible to determine $\beta - \alpha$ and also to determine on what edges the points *r*, *s*, $r'$ and $s'$ in the convergence set must lie. If the strength of convergence $\epsilon$ is not known then it will presumably not be possible to determine $\beta - \alpha$ as strong convergence acting for a shorter time period would appear equivalent to weaker convergence acting over a longer time period. However, in this case it may again be possible to at least localise which edges the points *r*, *s*, $r'$ and $s'$ occur on.

More generally, we have only considered the case of a single pair of convergent paths. In future work, it would be interesting to consider conditions under which multiple convergence events might be distinguishable from simple tree-like evolution. Even so, some care may need to be taken with choosing the number of parameters as there could be issues with overfitting [see (Steel 2005)], as well as our underlying assumption of clock-like evolution [see (Mitchell et al. 2018, p. 914) for related discussion]. Furthermore, in practice it would be useful to develop algorithms to return a phylogenetic tree along with pairs of sets of edges for which there is evidence of convergence operating given a distance matrix as input.

While this paper shows that it will not be possible to recover all convergence events (or even one convergence event) from an observed distance in general, the results suggest intriguing possibilities for algorithms that could at least recover some partial information. This could be particularly useful in cases where convergence events have had a small enough impact so that the input metric is still triplet preserving.

One approach that we hope to explore in future work is to develop a method based upon algorithmic variants of the Build algorithm which can construct phylogenetic trees from sets of triplets [see e.g. Semple and Steel (2000)]. More specifically, we would begin by computing an unweighted tree *T* from a collection of triplets inferred from an input distance *d* on a set *X*, after which we would set the height of any internal vertex of *T* to be half the maximum taken over all distances between all pairs of taxa whose least common ancestor is that vertex. The distance associated to this weighted tree, ${\mathcal {T}}$, then forms an ultrametric, $d_{{\mathcal {T}}}$, that is greater than or equal to the observed distance *d* for any pair in *X*. We would then look for a convergence scenario $({\mathcal {T}}, R,\epsilon )$ with the aim of minimising the discrepancies $d_{\epsilon }^{({\mathcal {T}},R)}(x,y) -d(x,y)$, $x,y \in X$. Note that, in general, we would expect some variation from *d* being an ultrametric just due to random sampling rather than convergence, so we would probably need to also define some threshold of improvement to control the addition of convergence events.

Finally, as noted in the introduction, reticulate processes can also lead to a break down in the divergence model for evolution. Interestingly, in Francis and Steel (2015, Theorem 5) it is shown that in case a special type of phylogenetic network called a horizontal gene transfer network has a single cycle, then the so-called average distance (Willson 2012) that it induces satisfy the four-point condition if and only if the arcs in the network satisfy certain specific conditions. This result has similarities to what we find in Theorem 3, and points to the fact that models of reticulate evolution can also lead to tree metrics. Thus, it will be important to develop approaches that will allow us to distinguish between distances that are generated by reticulate versus convergent evolution. However this may not always be mathematically possible, in which case, as suggested in Mitchell et al. (2018), it may be useful to consider additional biological or biogeographical information to help decide which model to employ.

## Data Availability

Data sharing not applicable to this article as no datasets were generated or analysed during the current study.
